# Adalimumab reduces hand bone loss in rheumatoid arthritis independent of clinical response: Subanalysis of the PREMIER study

**DOI:** 10.1186/1471-2474-12-54

**Published:** 2011-02-27

**Authors:** Mari Hoff, Tore K Kvien, Johan Kälvesten, Aake Elden, Arthur Kavanaugh, Glenn Haugeberg

**Affiliations:** 1 Department of Rheumatology, St. Olavs Hospital, 7006 Trondheim, Norway; 2 Faculty of Medicine, Norwegian University of Science and Technology, 7491 Trondheim, Norway; 3 Department of Rheumatology, Diakonhjemmet Hospital and Faculty of Medicine, University of Oslo, 0319 Oslo, Norway; 4 Sectra, SE-58330 Linköping, Sweden; 5 Abbott Laboratories, 1330 Fornebu, Oslo, Norway; 6 Center for Innovative Therapy, Rheumatology, University of California, San Diego, CA 92037, USA; 7 Department of Rheumatology, Sørlandet Hospital, 4604 Kristiansand S, Norway

## Abstract

**Background:**

Anti-TNF therapy has been shown to reduce radiographic joint damage in rheumatoid arthritis (RA) independent of clinical response. This has previously not been examined for periarticular bone loss, the other characteristic feature of bone involvement in RA.

The objective of this study was to examine if treatment with the TNF-α inhibitor adalimumab also could reduce periarticular bone loss in RA patients independent of disease activity.

**Methods:**

RA patients were recruited from the PREMIER study and included 214 patients treated with methotrexate (MTX) plus adalimumab and 188 patients treated with MTX monotherapy. Periarticular bone loss was assessed by digital X-ray radiogrammetry metacarpal cortical index (DXR-MCI). Change in DXR-MCI was evaluated in patients with different levels of clinical response, as assessed by changes in DAS28 score at 52 weeks and in mean C-reactive protein (CRP) levels during follow-up.

**Results:**

In the MTX group, there was a greater median DXR-MCI loss among patients with moderate and high disease activity compared to those in remission or with low disease activity (-3.3% vs. -2.2%, p = 0.01). In contrast, periarticular bone loss was independent of disease activity (-1.9% vs. -2.4%, p = 0.99) in the combination group. In the MTX group patients with a mean CRP of ≥ 10 mg/l lost significantly more DXR-MCI than patients with low CRP (-3.1% vs. -1.9%, p <0.01) whereas in the combination group no significant differences between the two CRP groups was seen (-2.4% vs. -2.0%, p = 0.48).

**Conclusion:**

Adalimumab in combination with MTX reduces periarticular bone loss independently of clinical response. These results support the hypothesis that TNF-α stimulates the osteoclast not only by the inflammatory pathway but do also have a direct effect on the osteoclast.

**Trial Registration:**

ClinicalTrials (NCT): NCT001195663

## Background

In rheumatoid arthritis (RA), bone damage on radiographs is visible as erosions and periarticular osteoporosis. Substantial data support that both erosions and osteoporosis in RA share a common cellular pathway which involves stimulation of the osteoclast. This osteoclast activation depends on stimulation from receptor activator of nuclear factor-κ ligand (RANKL) which binds to the receptor activator of nuclear factor-κ (RANK) on the osteoclast. The expression of RANKL is stimulated by pro-inflammatory cytokines (i.a. TNF-α, interleukin-1 (IL-1), IL-6 and IL-17). In addition recent data also suggest decreased osteoblast activation through the Wnt system [1].

In comparison to disease modifying anti-rheumatic drugs (DMARDs) including methotrexate (MTX), anti-TNF therapy has been shown to be superior in reducing the rate of both radiographic joint damage [2-4] and hand bone loss [5,6]. Recently, the rate of radiographic joint progression was reported to be reduced independent of a patient's clinical response to anti-TNF therapy [7,8]. This may suggest an additional positive effect of anti-TNF therapy on bone in RA independent of its anti-inflammatory effect. This has previously not been examined for periarticular bone loss.

The objective of this study was to examine if treatment with the TNF-α inhibitor adalimumab also could reduce periarticular bone loss in RA patients independent of disease activity.

## Methods

The PREMIER study cohort was used to examine the relationship between periarticular bone loss and clinical response in RA patients treated with MTX and anti TNF-therapy.

In this cohort, radiographic joint progression has recently been reported to be reduced independently of patients' clinical responses to anti-TNF therapy with adalimumab [7].

The clinical, radiographic and bone density data from this 2-year, multi-centre, double-blind, randomised controlled study has previously been described in detail [6,9] In short, the efficacy and safety of adalimumab plus MTX was compared with adalimumab monotherapy and with MTX monotherapy in 799 adult patients with early (< 3 years, mean disease duration 9.1 month), aggressive RA (inclusion criteria: ≥8 swollen joint; erythrocyte sedimentation rate ≥28 or C-reactive protein (CRP) ≥1.5 mg/dl; erosions or rheumatoid factor positive), who previously had not been treated with MTX [9].

Digital X-ray radiogrammetry (DXR) (Sectra, Linköping, Sweden) was used to measure hand metacarpal cortical index (MCI) on the same digitised hand X-rays used for assessment of radiographic joint damage. DXR-MCI is defined as the combined cortical thickness divided by the bone width and is a relative bone measure independent of bone size and bone length [10,11]. In the literature short-time in-vivo precision (CV%) has been reported to range from 0.31-0.64% for DXR-MCI [10,12,13].

DXR-BMD (def: *cxVPA_comb_x*(1-*p*), where c is a density constant, VPA is volume per area, and p is porosity) was intended to be the main outcome measure in this study. However, many radiographs could not be analysed for BMD because of unknown image resolution. The equation for DXR-BMD is based on volume per area and requires a known resolution. Thus, DXR-MCI, which is a relative measure less dependent of image resolution, was used as the primary outcome measure [6]. DXR-MCI has been shown to be highly correlated with hand bone mineral density, both measured as DXA hand and DXR-BMD [14]. Detailed information on the DXR-MCI measurement of the PREMIER RA cohort has previously been reported [6].

This sub analysis involved 188 patients from the MTX group and 214 patients from the combination group with available DXR data and disease activity measured by DAS28 at 52 weeks [15]. The combination group received adalimumab 40 mg subcutaneously every other week plus weekly oral MTX (rapidly increased to 20 mg/week), and the monotherapy group received weekly oral MTX plus placebo injections.

Disease activity was assessed by the disease activity score using a 28 joint count (DAS28) at 52 weeks. Levels achieved for DAS28 scores were stratified according to the EULAR improvement criteria [16] for remission (DAS28 ≤ 2.60), low disease activity (DAS28 2.61-3.20), moderate disease activity (DAS28 3.21-5.10), and high disease activity (DAS28> 5.10).

DXR-MCI bone loss at 52 weeks follow- up were compared for patients in remission and low disease activity vs. moderate and high disease activity according to treatment groups, as well as for patients in remission, low, moderate and high disease activity.

DXR-MCI loss dependent on inflammation, assessed by CRP during follow-up, was also analysed according to treatment groups. Mean CRP was calculated from values at baseline, 26 weeks and 52 weeks follow-up, and a mean CRP of <10 was defined as low inflammation.

### Statistics

Since the data were skewed, non-parametric analyses were conducted. No imputations were performed. Baseline values were compared between treatment groups with the Mann-Whitney method for continuous variables and the chi-squared method for categorical variables. DXR-MCI loss is described as negative values. Group analyses were performed with the Mann-Whitney method for two independent samples and Kruskall-Wallis for more than two independent samples. Spearman correlation analyses were conducted in an attempt to correlate changes in DXR-MCI with disease activity measured by DAS28. Analyses that came out with a p-value ≤ 0.05 were considered as statically significant.

### Study ethics

The PREMIER study was approved a central institutional review board or independent ethics committee at each participating site approved the study, and all patients provided written informed consent [9].

## Results

Baseline characteristics are shown in Table 1. There were no statistically significant differences between the MTX and the combination group.

**Table 1:** Baseline characteristics for the examined early rheumatoid arthritis patients. Results are given in mean (± standard deviation) for continuous variables and numbers (percentages) for categorical variables.

	Adalimumab + Methotrexate (N = 214)	Methotrexate Monotherapy (N = 188)
**Demographic characteristics**		
Age (years)	51.8 (14.3)	52.6 (13.2)
Female %	152 (71.0)	137 (72.9)
**Clinical characteristics**		
Disease duration (years)	0.7 (0.8)	0.8 (0.9)
Previously taken DMARDs %	69 (32.2)	56 (29.8)
Previously taken corticosteroids %	79 (36.9)	64 (34.0)
Tender joint count (0-66)	30.5 (14.6)	31.9 (14.3)
Swollen joint count (0-66)	21.4 (11.4)	22.5 (12.1)
C-reactive protein (mg/l)	40.2 (42.4)	40.4 (40.9)
HAQ (0-3)	1.5 (0.6)	1.5 (0.7)
DAS28	6.3 (0.9)	6.3 (0.9)
DXR-MCI (mg/cm^2^)	0.45 (0.09)	0.45 (0.09)

DMARDs = disease modifying anti-rheumatic drugs; HAQ = Health Assessment Questionnaire; DAS28 = 28-joint disease activity score; DXR = Digital X-ray radiogrammetry; MCI = metacarpal cortical index.

### DXR MCI loss and DAS28 level

**Figure 1:**
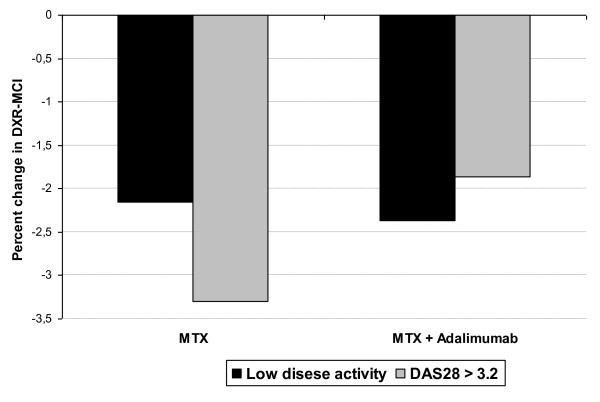
**DXR-MCI loss in the methotrexate (MTX) plus adalimumab and MTX groups dependent on disease activity measured by DAS28**.

In the MTX group there were a significantly greater DXR-MCI loss in the patients with elevated disease activity as assessed by DAS28 than patients in remission and low disease activity, median (interquartile range) (-3.3% (-6.0% to -1.4%) vs. -2.2% (-4.4% to -0.7%), p = 0.01). In the combination group there were no differences in DXR-MCI loss between patients with moderate and high disease activity vs. patient in remission or with low disease activity (-1.9% (-4.7% to -0.7%) vs. -2.4% (-4.1 to -0.5%), p = 0.99), and the levels of loss were closer to those observed for patients in the MTX group in remission (Figure 1).

Among the patients in remission or with low disease activity there were no difference between median DXR-MCI loss according to treatment groups (-2.4% in the combination group and -2.2% in the MTX group, p = 0.88). In the group who still had active disease, there was significantly greater median loss in the MTX group (-3.3% vs. -1.9%, p = 0.02) as compared to the combination group.

The DXR-MCI loss across subgroups in remission or with low, moderate and high disease activity is shown in Table 2. Among RA patients receiving MTX + adalimumab the bone loss was similar across the DAS28 subgroups (p = 0.97). For the MTX group there was a trend that the patients with high disease activity lost more DXR-MCI than patients with low disease activity (p = 0.10). The correlation (correlation coefficient r) between DAS28 and percentage DXR-MCI change was -0.14 (p = 0.06) in the MTX group and -0.07 (p = 0.33) for the combination group.

**Table 2:** DXR-MCI loss in the methotrexate plus adalimumab group and the MTX group stratified for disease activity measured by DAS28

	Adalimumab + methotrexate		Methotrexate	
	N = 214	Median (mean) % DXR-MCI change	N = 188	Median (mean) % DXR-MCI change
**Remission** **DAS28 ≤2.60**	118	-2.43 (-2.73)	53	-2.15 (-3.03)
**Low** **disease activity** **DAS28 2.61-3.20**	36	-1.98 (-2.59)	30	-2.09 (-2.90)
**Moderate** **disease activity** **DAS 28 3.21-5.10**	51	-2.01 (-3.17)	81	-3.33 (-4.65)
**High** **disease activity** **DAS28> 5.10**	9	-1.63 (-2.72)	24	-3.02 (-4.64)

DAS28 = 28-joint disease activity score; DXR = Digital X-ray radiogrammetry; MCI = metacarpal cortical index

### DXR MCI loss and CRP level

**Figure 2:**
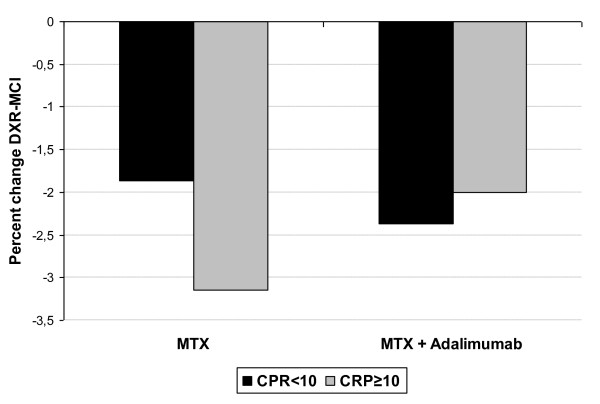
**DXR-MCI loss in the methotrexate (MTX) plus adalimumab and MTX groups according to CRP level achieved**.

In the MTX group 184 patients had CRP measures at baseline, 26 and 52 weeks and in the combination group the number was 206. In the MTX group there were 57 patients with mean CRP < 10 mg/L and 127 with CRP ≥10 mg/L. In the combination group the respective values were 86 and 120. The median DXR-MCI loss in the MTX group was significantly higher among patients with high CRP than low CRP (-3.1% (-6.0 to -1.4) vs. -1.9% (-3.9 to -0.2), p <0.01). In the combination group there was no difference regarding median DXR-MCI loss dependent on CRP level (-2.4% (-4.6% to -0.7%) vs. -2.0% (-4.1% to 0.8%), p = 0.48), Figure 2. The patients with high CRP treated with MTX lost significant more DXR-MCI than patients treated with MTX and adalimumab (-3.1 vs. - 2.4, p = 0.02), while there was no difference in bone loss in patient with low CRP (- 1.9 vs. -2.0, p = 0.78).

## Discussion

The main finding in this study was that adalimumab in combination with MTX reduces hand bone loss independently of clinically assessed disease activity. This has previously been shown for radiographic joint damage [7,8], but the present study is the first to show this for hand bone loss. This disconnection between inflammation and bone loss was not seen in MTX-treated patient. Patients with a poor clinical response or elevated CRP are at high risk of developing bone damage when treated with MTX monotherapy, while the bone will be protected independent of clinical response among users of MTX in combination with anti-TNF-α therapy.

These results support the hypothesis that TNF-α can influence bone loss not only by stimulating RANKL by inflammation. The most probable additive mechanism is that TNF-α activates the osteoclast directly by binding to osteoclasts precursors through TNF-α receptor. Blocking of this receptor will down regulate the osteoclast formation [1,17]. Another theoretical mechanism of TNF-α could be inflammation independent stimulation of RANKL, however this has not yet been clinically proven. The fact that TNF-α does have more than one way of stimulating the osteoclast may explain the positive effect on bone despite any clinical improvement [7,8].

Despite that bone loss was independent of disease activity in the combination group, there were still a bone loss observed. This loss may have been a result of the patient selection in the PREMIER study. The included patients had high disease activity and poor prognosis in terms of bone damage as Rheumatoid Factor-positive and erosive disease [9,18].

This is a retrospective study and we did not have any influence on the technical conditions regarding the radiographs. Due to difficulties analysing DXR-BMD the relative measure DXR-MCI was used, as described in detail previously [6]. However, DXR has improved the precision of MCI, and there is a strong correlation between DXR-BMD and DXR-MCI (r > 0.9) [6,14]. Another limitation was our inability to retrieve information on the use of bisphosphonates. This may be of importance as treatment with bisphosphonates increases DXR bone density [19].

## Conclusion

We conclude that adalimumab in combination with MTX reduces hand bone loss independent of disease activity. This study highlights the bone protecting effect of anti-TNF-α therapy.

## List of abbreviations

BMD: bone mineral density; CRP: C-reactive protein; DAS28: disease activity score based on 28 joint count; DMARDs: disease modifying anti-rheumatic drugs; DXR: digital X-ray radiogrammetry; ESR: erythrocyte sedimentation rate; HAQ: health assessment questionnaire; IL: interleukin; MCI: metacarpal cortical index; MTX: methotrexate; RA: rheumatoid arthritis; RANK: receptor activator of nuclear factor-κ; RANKL: receptor activator of nuclear factor-κ ligand; TNFα: tumour necrosis factor alpha

## Competing interests

M. Hoff, T. K. Kvien, A. Kavanaugh and G. Haugeberg have received consulting fees as speakers from Abbott Laboratories. Tore K. Kvien, A. Kavanaugh and G. Haugeberg have received founding for independent research from Abbott Laboratories. Aake Elden is employed by Abbott Laboratories. Johan Kälvesten is employed by Sectra.

## Authors' contributions

MH performed statistical analyses and prepared the manuscript. TKK contributed to statistical analysis and interpretation of results. JK analysed the radiographs. AE prepared the collection of radiographs. AK contributed to statistical analysis and interpretation of results. GH contributed to statistical analysis and interpretation of results and was the main responsible investigator of this project. All the authors substantially contributed to the manuscript. All authors read and approved the final manuscript.

## Pre-publication history

The pre-publication history for this paper can be accessed here:

http://www.biomedcentral.com/1471-2474/12/54/prepub
